# Impact of a community-based primary healthcare programme on childhood diphtheria-tetanus-pertussis (DPT3) immunisation coverage in rural northern Ghana

**DOI:** 10.1007/s43999-023-00032-8

**Published:** 2023-12-05

**Authors:** Edmund Wedam Kanmiki, Abdullah A. Mamun, James F. Phillips, Martin O’Flaherty

**Affiliations:** 1 Institute for Social Science Research, The University of Queensland, Indooroopilly, QLD 4068 Australia; 2 Poche Centre for Indigenous Health, Faculty of Health and Behavioural Sciences, The University of Queensland, Brisbane, Australia; 3 ARC Centre of Excellence for Children and Families Over the Life Course (The Life Course Centre), The University of Queensland, Indooroopilly, QLD 4068 Australia; 4 Heilbrunn Department of Population and Family Health, Mailman School of Public Health, Columbia University, New York, NY USA

**Keywords:** Child health services, Community-based programmes, Immunisation coverage

## Abstract

**Background:**

Child healthcare services such as diphtheria-tetanus-pertussis (DPT3) vaccination are known to reduce childhood mortality and morbidity. However, inequalities in access to these services in developing countries continue to constrain global efforts aimed at improving child health. This study examines the impact and equity effect of a community-based primary healthcare programme known as the Ghana Essential Health Intervention Programme (GEHIP) on improving the uptake of childhood DPT3 immunisation coverage in a remote rural region of Ghana.

**Methods:**

Using baseline and end-line household survey data collected from mothers, the effect of GEHIP’s community-based healthcare programme on DPT3 immunisation coverage is evaluated using difference-in-differences multivariate logistic regression models. Household wealth index and maternal educational attainment were used as equity measures.

**Results:**

At end-line, both intervention and comparison districts recorded increases in DPT3 immunisation coverage although intervention districts had a relatively higher coverage than comparison districts (90% versus 88%). While children resident in intervention areas had slightly higher rates than children resident in comparison areas, regression results show that this difference was not statistically significant (DiD = 0.038, *p*-value = 0.102). There were also no significant equity disparities in the coverage of DPT3 vaccination for both household wealth index and maternal educational attainment.

**Conclusion:**

DPT3 vaccination coverage in both study arms met the global vaccine action plan targets. However, because estimated effects are not significantly higher among treatment area children than among comparison districts counterparts, no equity/inequity effects of the community-based healthcare programme on DPT 3 coverage is evident.

**Supplementary Information:**

The online version contains supplementary material available at 10.1007/s43999-023-00032-8.

## Background

Globally, significant progress has been made in the reduction of under-five child mortality. Between the years 1990 and 2020, global under-five mortality reduced by about 61% (from 93 per 1,000 to 37 per 1,000 deaths) [1]. Despite this, improving child survival is still a matter of great concern. It is estimated that in the year 2020 about 13,800 under-five deaths occurred each day on average [1]. It is further projected that between 2017 and 2030, about 10 million children will lose their lives from preventable causes before reaching their fifth birthday [2].

Sub-Saharan Africa alone accounts for more than 50% of global under-five deaths [3]. In Ghana, despite considerable efforts, under-five mortality is high at 44 per 1000 live births [1]. It is higher in poor rural areas compared to urban settings [4]. The current Sustainable Development Goals (SDGs) aim to reduce global under-five mortality to less than 25 per 1000 live births by 2030 [5]. These are quite ambitious given the prevailing rates of reduction, in Ghana, this requires about a fifty per cent reduction in under-five mortality.

Access to antenatal and postnatal health services including immunizations has proven to reduce the incidence of mortality for both mothers and children [2, 6, 7], For instance, measles vaccination alone is estimated to have averted about 15.6 million child deaths between 2000 and 2015 [8]. But only half of the women and children in developing countries receive the recommended amount of care during pregnancy, birth and postnatal periods [8].

The Global Vaccine Action Plan (GVAP) endorsed by World Health Assembly in 2012 called for all countries to increase the coverage of all childhood immunizations in their national immunization schedules to 90% national coverage by 2020 [9]. Noting the high inequality gaps that exist between and within countries, the GVAP called for a focus on improving equity in vaccine coverage between low-income countries and high-income countries as well as at the sub-national levels [9]. Unfortunately, although there has been some global progress towards attaining the GVAP targets for several vaccines, disparities between and within countries continue to persist [10] The global burden of disease study found that in sub-Saharan Africa, only 26% of countries met the target for DPT3 coverage, 33% for Measles-containing-vaccine first-dose (MCV1), 17% for polio and 26% for both Hepatitis B(HepB3) and Haemophilus influenzae type b (Hib3) met targets respectively [11]. With wide disparities persisting within countries [10].

Inequalities in access to and use of maternal and child health services expose millions of women and children from poor households to the risk of preventable diseases and deaths [2]. It has been observed that poor child health is intertwined with poverty and economic inequality [11]. Evidence shows that there exist inequalities in maternal and child health across socio-economic strata, which is linked to inequities in access to healthcare services [12, 13]. The United Nations for instance reports that children born to poor households are twice as likely to die before their fifth birthday compared to those born to wealthier households, and those born to educated mothers are also better off than those born to mothers with no formal education [8]. Often, the poor and low educated lack the ability and capacity to navigate modern health systems for care and are less likely to be able to afford health services, optimal nutrition and sanitation required for good maternal and child health [14].

Community-based primary healthcare programmes have emerged as low-cost approaches for improving healthcare access and reducing inequalities in maternal and child healthcare [15]. This paper assessed the effectiveness of a community-based primary healthcare programme on DPT3 immunisation coverage. The importance of evaluating the equity effects of community-based care is fundamental to help identify and design low-cost but effective strategies for improving maternal and child health in the context of low-and middle-income countries (LMICs).

### Community-based healthcare programmes

Community-based healthcare programmes are strategies for delivering basic healthcare services. In rural remote communities, they have been recognised as culturally safe approaches to delivering low-cost essential healthcare. Evidence suggests that community-based programmes can help reduce health inequalities in rural poor communities through the reduction of barriers to seeking healthcare [15, 16]. In Ghana, research found that when properly trained community health workers are deployed with requisite logistics in communities, their work can have health equity effects on childhood mortality [17]. However, these equity improvements were conditional on communities being allocated with trained community health nurses assisted by community volunteers. Communities that were allocated with community volunteers alone with no clinical training experienced no equity effects [17]. While community volunteers are able to support primary healthcare delivery in rural communities by assisting nurses with social mobilisation and rudimentary health promotion activities, their work alone leads to minimal health benefits unless it is combined with a clinically trained nurse with capacity for delivering primary preventive and curative healthcare at the community level [17].

There is growing evidence that maternal and child healthcare delivery through community-based health programmes in remote communities improves health outcomes including healthy lifestyle practices and health-seeking behaviour leading to improved maternal and newborn health in rural communities [18–20]. Although this approach to service delivery has considerable promise for reducing mortality and morbidity in these rural communities [18], evidence of the ability of community-based health programmes impact on equity in service delivery is sparse. A systematic review found that community-based programmes improve access to care but could not find evidence that they improve equity in services received [21].There is therefore the need for research examining the equity effect of community-based health programmes.

Poor and disadvantaged people face several barriers to healthcare access including the cost of care, illiteracy, poor transportation, shortage of health workers, low knowledge of services and care practices, and religious as well as cultural beliefs and practices among others [2, 12, 18]. An important step is to understand policy mechanisms that can effectively overcome these barriers and improve health equity. Further studies have been recommended to provide evidence on how best health systems can meet the health needs of newborns in socio-culturally responsive and economically feasible ways [22].

Community-based healthcare programmes are known to reduce barriers to healthcare delivery in a culturally responsive way leading to improve service delivery for rural poor communities, this could also improve childhood immunization coverage and have positive equity effects. This study contributes to understanding the effect of Ghana Essential Health Interventions Programme (GEHIP)’s community-based health systems strengthening programme on improving equity in immunization coverage. The goal is to help identify strategies with high potential for improving equitable delivery of child health services.

## Methods and materials

### Study context

In Ghana, a primary healthcare programme called the Community-based Health Planning and Service Programme (CHPS) was developed and made a national programme for improving access to healthcare in rural remote settings of the country in the year 2000. This was after field trials and experimentations that demonstrated that community-based healthcare improves access to and use of primary healthcare for rural communities leading to improvements in several maternal and child health indicators [23–25]. Unfortunately, after a decade of nationwide policy was announced, CHPS national scale-up was slow owing to the persistence of operational constraints imposed by technical, financial, logistical and leadership capacity limitations. To help address these challenges and demonstrate practical ways of scaling up CHPS and its functioning, the Ghana Essential Health Interventions Programme (GEHIP) was implemented in districts of the Upper East Region, which represented one of the most remote regions of Ghana. GEHIP focussed on strengthening primary health care using the six WHO health system building blocks: leadership development and planning, human resources capacity building, health information for decision–making, health service delivery, essential medicine and vaccines and healthcare financing improvement. In addition, community and district-level stakeholder engagement were key to engendering grass-root support for galvanizing local resources for CHPS functioning.

GEHIP was a five-year implementation research programme implemented in the Upper East region of northern Ghana between 2010–2015. Three districts were the focus of the intervention while four other contiguous districts served as comparison districts for evaluation purposes. Both intervention and comparison districts are similar in terms of their socioeconomic deprivation and geographic isolation [26]. The Upper East region has a population of about 1.3 million, It is characterized by pervasive poverty (55% prevalence) and low formal educational attainment (only 30.9% of the adult population around the time of the intervention [27–30]. The project was implemented in this rather challenging socioeconomic environment to enhance the policy relevance of any success that might result from its implementation. Previous studies have demonstrated that GEHIP expanded community-based primary healthcare leading to improvement in some health outcomes [27, 31, 32]. However, previous work has not investigated the consequences of the GEHIP programme for health service equity as it relates to childhood immunization coverage.

### Source of data

To address these research questions, baseline and end-line survey data from GEHIP is used in this study. These surveys were part of the project evaluation approach. Information collected during the survey included demographic and socio-economic characteristics of mothers and their households, birth histories, health care utilisation, antenatal care, and postnatal care including childhood immunisation. The sampling design was a multi-stage process in which 66 predominantly rural Enumeration Areas (EAs) were selected at the first stage followed by household sampling proportional to the population size of each EA. Within each sampled household, all women of reproductive age were eligible to be interviewed [32]. The baseline survey was conducted in 2010/2011 while the endline survey took place in 2014/2015.

The same EAs were used for both baseline and endline however, no effort was made to interview the same women or link data from baseline to endline of same women. A total of 5604 women were interviewed at baseline and 5914 were interview at endline. This paper uses a subset of this women who had recent births and provided information on their children vaccination coverage status. Analysis in this paper is restricted to women whose recent birth was at least one year old at the time of interview to ensure that they were passed the aged to receive all three doses of DPT3 immunisation. An evaluation of GEHIP showed that it led to about 48% reduction in neonatal mortality in implemented districts over and above reductions observed in comparison districts [32]. However, the extent to which this community-based health programme impacted on equitable care and health outcomes is unknown. This current study uses household survey data collected from reproductive-aged women at baseline and end line of GEHIP to answer critical questions on the equity effect of GEHIP on childhood vaccination coverage. After accounting for this restriction and the availability of DPT3 immunisation records, our sample size included 2,812 women made up of a baseline sub-sample of 1,205 women and end-line sub-sample includes 1,607 women.

### Data analysis

The main outcome of interest is receiving at least three doses of Diphtheria, Pertussis (whooping cough), and Tetanus vaccination (DPT3). A child who received the full DPT 3 vaccination was coded 1 and 0 if did not receive the vaccine or were only partially vaccinated. Information on vaccination status was collected from the child health book provided by study participants. Data collectors also used follow-up questions to clarify situations in instances where services might have been provided but records not made in the child’s health book or those not able to provide a health book. For those without a health book or no record of DPT 3 in their health book, data collectors described how DPT3 is given to help the mother or caretaker identify if the child was administered the vaccine. This analysis is restricted to respondents whose recent birth is at least one year old to ensure that their children were old enough to have received three doses of DPT 3.

The household wealth index and maternal educational attainment were the two main proxy measures of socioeconomic status used in analysing inequalities. Maternal educational status was categorised into three: no formal education, primary/junior high school and secondary/above. In regression analysis, control variables included in models are maternal age, marital status, rural/urban location of residence, parity, and education. To test the effect of GEHIP’s community-based health programme on DPT 3 vaccination status, the Heckman difference-in-differences regression is used to estimate the average treatment effect of the intervention [33]. The difference in difference equation is given as:

$${{\varvec{L}}{\varvec{o}}{\varvec{g}}{\varvec{i}}{\varvec{t}}{\varvec{Y}}}_{{\varvec{i}}{\varvec{g}}{\varvec{t}}}\boldsymbol{ }=\boldsymbol{ }{{\varvec{\beta}}}_{0}\boldsymbol{ }\boldsymbol{ }+\boldsymbol{ }{{\varvec{\theta}}}_{{\varvec{t}}}\boldsymbol{ }+\boldsymbol{ }{{\varvec{\beta}}}_{1}{\varvec{G}}+\boldsymbol{ }{{\varvec{\beta}}}_{2}{\varvec{t}}\boldsymbol{ }+\boldsymbol{ }{{\varvec{\beta}}}_{3}{\varvec{G}}.{\varvec{t}}\boldsymbol{ }+\boldsymbol{ }{{\varvec{U}}}_{{\varvec{i}}{\varvec{g}}{\varvec{t}}\boldsymbol{ }}+\boldsymbol{ }{{\varvec{\varepsilon}}}_{{\varvec{i}}{\varvec{g}}{\varvec{t}}}$$

Here, Y represents the DPT3 immunisation status. ${{\varvec{\upbeta}}}_{0}$ is the fixed effects of the baseline group observations, otherwise known at the intercept. The period time-invariant effects are captured by ${{\varvec{\uptheta}}}_{t}$_**.**_ G represents treatment status, G = 1 for observations from the treatment districts and G = 0 for those in non-treatment districts. t is an indicator variable for baseline (= 0) or endline/ (= 1) measurements, the ${{\varvec{\upbeta}}}_{\mathrm{s}}$ are the regression coefficients to be estimated; U_igt_ captures individual-level factors that predict DPT3 immunisation. Predictor variables include the mother’s age, marital status, educational status, household wealth index, religion and parity. **ε**_***igt***_ is the error term.

In addition, a binary logistic regression with two and three-way interaction terms is used to estimate the GEHIP’s programme equity effects on DPT 3 vaccination coverage using household wealth index and maternal educational status as equity measures. The equation is represented by;

$${\varvec{L}}{\varvec{o}}{\varvec{g}}{\varvec{i}}{\varvec{t}}({{\varvec{Y}}}_{{\varvec{i}}{\varvec{j}}})\boldsymbol{ }=\boldsymbol{ }{{\varvec{\beta}}}_{0}+\boldsymbol{ }{{\varvec{\beta}}}_{1}{\varvec{G}}\boldsymbol{ }+\boldsymbol{ }{{\varvec{\beta}}}_{2}\boldsymbol{ }{\varvec{T}}\boldsymbol{ }+\boldsymbol{ }{{\varvec{\beta}}}_{3}({{\varvec{G}}}^{\boldsymbol{*}}{\varvec{T}})+\boldsymbol{ }\boldsymbol{ }{{\varvec{\gamma}}}_{1}\boldsymbol{ }{\varvec{W}}+\boldsymbol{ }\boldsymbol{ }{{\varvec{\gamma}}}_{2}({{\varvec{W}}}^{\boldsymbol{*}}{\varvec{T}})\boldsymbol{ }+\boldsymbol{ }\boldsymbol{ }{{\varvec{\gamma}}}_{3}\boldsymbol{ }(\boldsymbol{ }{{\varvec{G}}}^{\boldsymbol{*}}\boldsymbol{ }{\varvec{W}})\boldsymbol{ }+\boldsymbol{ }{\varvec{\gamma}}4({{\varvec{W}}}^{\boldsymbol{*}}\boldsymbol{ }{{\varvec{G}}}^{\boldsymbol{*}}\boldsymbol{ }{\varvec{T}})\boldsymbol{ }+\sum\nolimits_{{\varvec{j}}=1}^{{\varvec{J}}}{{\varvec{\delta}}}_{{\varvec{j}}}{{\varvec{x}}}_{{\varvec{j}}}$$

Where, ***Y*** is the binary outcome indicator of an individual i at time t (taking the values 1 and 0). **G** is an area indicator for treatment districts (G = 1) and zero otherwise. $\mathbf{T}$ is a dummy variable defining survey time, T = 1 for the end line and 0 for baseline observations. ${{\varvec{\beta}}}_{0}$ is the intercept, ${{\varvec{\upbeta}}}_{\mathbf{s}}$ are the regression coefficients to be estimated by maximum likelihood. $\mathbf{W}$ is the household wealth index. The parameters ${{\varvec{\upgamma}}}_{1}, {{\varvec{\upgamma}}}_{2}$, and ${{\varvec{\upgamma}}}_{3}$ represent adjusted effects of wealth in comparison districts at baseline, the change in the effect of wealth in comparison districts between baseline and end-line, and the difference in the effect of wealth between intervention and comparison districts at baseline respectively. Thus, ${{\varvec{\upgamma}}}_{4}$ estimates the effect of GEHIP on health equity relative to comparison districts, that is the difference in change in equity between intervention and comparison districts. The vector $\mathbf{X}$ refers to $\mathbf{J}$ control variables in the model. STATA 16 software was used in all the analysis.

## Results

Table 1 presents the background statistics of the study sub sample. The were generally similar between the two study arms. However, at baseline, only about 2.3% were teenagers while 5.1 were teenagers at end-line for both intervention and comparison groups. Respondents between 20–34 years old were the majority at baseline (~ 63% and 67% for intervention and comparison respectively) and at end line, 64% and 70% respectively. About 92% of respondents were reported to be married at both baseline and end-line. Respondents with no formal education were as high as 70% and 77% at baseline for intervention and comparison respectively and this dropped to 64% and 69% respectively at end-line, indicating a growing trend in formal education. Christianity was the most common religion as 49% and 56% reported to be affiliated with Christianity at baseline and endline respectively. Respondents associated with Islamic religion also increase from 24 and 34% at baseline to 30% and 32% at endline. This shows that the proportion of Christians and Muslims and growing in the study area while African traditional religion is reducing (from ~ 21% at baseline to ~ 12% at endline).

**Table 1:** Background characteristics of study sample

**Descriptive Statistics**		**Baseline Survey**				**End line Survey**			
		**Intervention**		**Comparison**		**Intervention**		**Comparison**	
		**N**	**%**	**n**	**%**	**n**	**%**	**n**	**%**
**Age Group**	15–19	14	2.3	12	2.1	42	5.1	40	5.1
	20–34	384	63.2	390	67.4	532	64.5	547	70.0
	35–49	210	34.5	177	30.6	251	30.4	195	24.9
**Marital Status**	Single	44	7.2	39	6.6	68	8.2	66	8.4
	Married	569	92.8	553	93.4	757	91.8	716	91.6
**Education**	No formal education	427	69.8	456	77.2	530	64.2	543	69.4
	Prim/JHS/middle sch	161	26.3	120	20.3	235	28.5	198	25.3
	Sec/tertiary	24	3.9	15	2.5	60	7.3	41	5.2
**Religion**	Christianity	332	54.2	257	43.4	466	56.5	430	55.0
	Traditional	132	21.5	133	22.5	106	12.9	100	12.8
	Islam	149	24.3	202	34.1	253	30.7	252	32.2
	Buli	212	34.6	1	0.2	259	31.4	1	0.1
**Ethnicity**	Frafra	146	23.8	242	40.9	162	19.6	370	47.3
	Kusasi	123	20.1	240	40.5	176	21.3	259	33.1
	Other	132	21.5	109	18.4	228	27.6	152	19.4
**Location of Residence**	Urban	11	1.8	13	2.2	116	14.1	25	3.2
	Semi-urban	86	14.0	23	3.9	102	12.4	142	18.2
	Rural	516	84.2	554	93.9	607	73.6	615	78.6
**Wealth Index (5)**	Poorest	159	25.9	99	16.8	170	20.6	118	15.1
	Poorer	133	21.7	106	18.0	164	19.9	143	18.3
	Better	95	15.5	127	21.6	197	23.9	156	20.0
	Less Poor	129	21.0	129	21.9	164	19.9	159	20.3
	Least Poor	97	15.8	127	21.6	130	15.8	206	26.3
**Parity**	One birth	100	16.3	101	17.1	178	21.6	193	24.7
	2–4 births	288	47.0	296	50.0	396	48.0	350	44.8
	5 or more	225	36.7	195	32.9	251	30.4	239	30.6

 Table 2 shows the weighted percentage coverage of DPT3 vaccination by intervention and comparison districts. DPT3 vaccination coverage increased slightly between baseline and end line for intervention districts but non-intervention districts remain relatively unchanged. The general high coverage in both arms of the study however, is indicative of broader efforts by national and regional health authorities towards improving child health in the study region Table 2.

**Table 2:** DPT3 vaccination coverage by intervention and comparison districts

	Unweight Coverage		Weighted Coverage	
	% Coverage	95% CI	% Coverage	95% CI
Intervention baseline	90.20	88.90 – 91.49	90.10	88.75 – 91.40
Non-Intervention baseline Survey	88.32	86.91 – 89.74	88.49	87.08 – 89.90
Intervention end-line	91.52	90.01 – 92.96	91.27	89.76 – 93.77
Non-intervention end-line	88.43	86.73 – 90.12	88.62	86.94- 90.30

Table 3 presents results of the difference-in-difference regression estimates controlling for covariates. The results show that although there was a significant improvement in DPT 3 vaccination coverage at endline, the average treatment effect of GEHIP on DPT3 vaccination coverage is not statistically significant (DiD = 0.038, p-value = 0.102). Thus, GEHIP did not contribute to significant improvement in DPT3 vaccination coverage relative to the comparison areas.

**Table 3:** Difference-in-difference estimations

Outcome var	DPT Vaccination	S. Err	t	*P* > t
**Before**				
Control	0.862			
Treated	0.870			
Diff (T-C)	0.008	0.019	0.39	0.693
**After**				
Control	0.886			
Treated	0.932			
Diff (T-C)	0.046	0.014	3.25	0.001***
**Diff-in-Diff**	0.038	0.023	1.63	0.102

R-square: 0.01, * Means and Standard Errors are estimated by linear regression, **Robust Std. Errors, **Inference: *** *p* < 0.01; ** *p* < 0.05; * *p* < 0.1

**Fig. 1:**
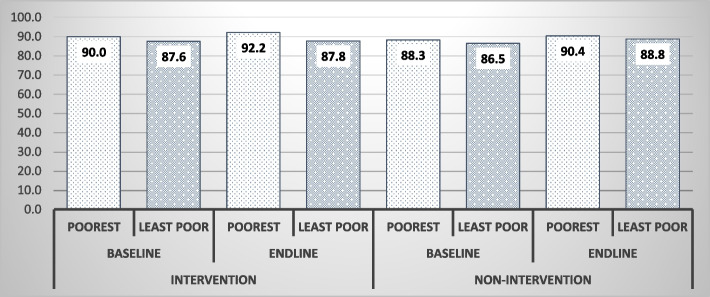
DPT3 Vaccination coverage by poorest and least poor wealth quintile

Figure 1 shows a comparison of the percentage coverage of DPT3 vaccination between the poorest wealth quintile and the least poor quintile by intervention and non-intervention areas. Within the intervention group, respondents in the poorest quintile increased from 90 to 92% while those in the least poor quintile were relatively unchanged. In the non-intervention group, both wealth quintiles increased by approximately 2%.

**Fig. 2:**
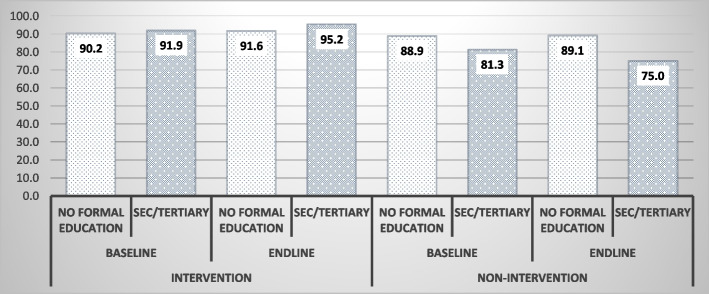
DPT3 Vaccination coverage by maternal education

Figure 2 shows the comparison of DPT3 vaccination coverage among respondents with no formal education to those with secondary education or above. In the intervention group, respondents with no formal education increased by just 1% (from 90.2% to 91.6%) while those with up to secondary educational attainment increased by about 4%. In the non-intervention group however, respondents with no formal education had almost the same coverage rate for baseline and endline while those with secondary education or above rather observed a reduction from 81 to 75%.

Table 4 presents the average marginal effects of household wealth index and maternal educational attainment from regression analysis. The full regressions can be found in supplementary Tables S1 and S2. Contrary to expectation, results indicate that there was no statistically significant marginal effect of both household wealth index and maternal educational attainment on the coverage of DPT 3 vaccination at both baseline and endline.

**Table 4:** Average marginal effect of wealth and education on DPT 3 coverage

Delta-method						
	**dy/dx**	**std. err**	**z**	***P* > z**	**[95% conf**	**interval]**
**Average marginal effect of household wealth on DPT coverage**						
Baseline Non-intervention group	0.012	0.011	1.020	0.308	-0.011	0.034
Baseline Intervention group	-0.014	0.009	-1.520	0.128	-0.032	0.004
End line Non-intervention group	-0.006	0.009	-0.680	0.499	-0.025	0.012
End line Intervention group	-0.006	0.008	-0.790	0.431	-0.021	0.009
**Average marginal effect of maternal education**						
Baseline Non-intervention group	-0.010	0.024	-0.430	0.670	-0.058	0.038
Baseline Intervention group	0.018	0.025	0.710	0.478	-0.032	0.068
End line Non-intervention group	-0.028	0.021	-1.320	0.186	-0.070	0.014
End line Intervention group	0.008	0.017	0.500	0.614	-0.024	0.041

## Discussion

This study assessed the equity effect of GEHIP’s community-based healthcare programme on DPT3 immunization coverage given changes associated with GEHIP intervention. Results show generally high coverage rates of DPT3 vaccination in the study area. Yet rates observed in intervention districts increased from 90% coverage to 91% and corresponding coverage rates in comparison districts remaining relatively unchanged (88%). This modest differential associated with GEHIP intervention districts was not statistically significant. As of the year 2015 when the endline survey was conducted, the national DPT 3 coverage rate was 88%, a level that was similar to GEHIP treatment and comparison study districts [34].

Difference in difference results after controlling of potential confounders such as respondent’s age, marital status, religious affiliation, location of residence (rural/urban) and parity, show that coverage rates of DPT3 immunizations for GEHIP treatment and comparison areas differences were not statistically significant. Equity analysis results are similar. In the univariate analysis, respondents in the poorest socioeconomic status and those with no formal education in intervention districts observed more improvements in vaccination coverage compared to similar groups in the non-intervention districts. However, controlling for confounders in regression analysis, there was no statistically significant equity/inequity effects of GEHIP using both household wealth and maternal education as equity measures. DPT3 immunization coverage is one of the priority programmes that the WHO and other international agencies provide support for countries in west Africa thus there is no fee at the point of service [35]. This suggests that both intervention and comparison districts equivalently benefited from this existing support from the Global Vaccine Action Plan (GVAP), with results achieving the target of ~ 90% coverage [9]

Although the results of the effect of GEHIP on DPT3 vaccination coverage appear to be counter intuitive, our null findings reflect the nationwide efforts put by the Ghana health service and its multinational and national partners towards improving childhood immunization in the country. A careful reflection on the context and background to GEHIP’s implementation also provides important leads to understanding these results. Firstly, there was a nationwide newborn healthcare programme whose implementation involved both GEHIP’s intervention and non-intervention districts.

In 2000, Ghana adopted CHPS as its core implementation strategy for achieving universal access to primary health care. When evidence showed that this program was progressing too slowly, GEHIP was fielded to test means of accelerating CHPS implementation. Monitoring showed that only 25% of study area communities were equipped with CHPS facilities in the GEHIP intervention districts at baseline. This coverage increased to 50% in the comparison area and 100% in GEHIP treatment areas. While this expansion of community facility accesses improved primary care coverage and reduced childhood mortality, GEHIP was launched in the context of successful implementation of the national EPI programme that employed outreach strategies that were independent of CHPS facilities. The success of the DPT3 coverage activities were mainly independent of the added value of GEHIP implementation. The modest acceleration of DPT3 coverage may have been associated with GEHIP strategies, but statistical analysis shows that this posited effect was not significant.

Results of this study are in line with a previous study of 14 West African countries showing that Ghana was among the top three West African countries in achieving the goal of equitable coverage of DPT 3 and skilled birth attendance relative to other countries in the region, such as Nigeria, Guinea and Niger [35].

### Study limitations

Both study arms recorded high immunisation coverage of DPT3 thereby approaching levels specified by global targets. With such high coverage rates, the possibility of detecting inequity or reducing inequity is low. Also, given the proximity of intervention and control districts and the fact that both study arms where under the same regional director of health services and held joint annual review meetings, there are concerns about potential spillover of interventions. Nonetheless, this investigation is warranted by the need to determine if achieving universal CHPS coverage added to the progress that remained to be achieved for DPT3 coverage to be complete, at last. This study was conducted in one of the poorest regions in Ghana and thus our results on equity may potentially be masked by the general pervasive poverty in the study setting. The above notwithstanding, this paper has contributed to the evidence based on the potential contribution of community-based primary health programmes on childhood immunisation coverage.

## Conclusion

This study found improvements in DPT 3 immunisation coverage in both arms of the study. Both areas meet the GVAP targets of 90%. Although GEHIP intervention led to slightly higher coverage rates than comparison districts, these were not statistically significant as both were very high rates. Also, no equity/inequity effects were found due to the high coverage rates. There is a need for Ghana health service and its partners to continue their efforts so as to sustain the gains with regard to DPT 3 immunisation coverage and all the other maternal and child healthcare services in order to accelerate the phase towards achieving the sustainable development goals. Future research should continue to track progress across different sections of the population to ensure that vulnerable sections are not left behind.

### Supplementary Information

**Additional file 1: Table S1.** Logistic Regression Results of Effect of Wealth on DTP3 Vaccination Coverage. **Table S2.** Logistic Regression Results of the effect of Education on DTP3 Vaccination Coverage.

## Data Availability

All data upon which this study is based can be obtained from the authors upon request.
