# Supplementary material for: Impact of a community-based primary healthcare programme on childhood diphtheria-tetanus-pertussis (DPT3) immunisation coverage in rural northern Ghana
Source: Res Health Serv Reg. 2023 Dec 5;2:18. doi: 10.1007/s43999-023-00032-8 (PMC11281747; doi:10.1007/s43999-023-00032-8)
Supplement: Supplementary file 1 — Additional file 1: Table S1. Logistic Regression Results of Effect of Wealth on DTP3 Vaccination Coverage. Table S2. Logistic Regression Results of the effect of Education on DTP3 Vaccination Coverage. [file 43999_2023_32_MOESM1_ESM.docx]

**Supplementary Tables**

**Table S1: Logistic Regression Results of Effect of Wealth on DTP3 Vaccination Coverage**

| **VARIABLES** | **OR** | **95% CI** |
| --- | --- | --- |
|  | | |
| Wealth | 1.11 | (0.91 - 1.37) |
| 1.Treatment | 2.21* | (0.93 - 5.25) |
| 0b. Treatment #co. Wealth | 1.00 | (1.00 - 1.00) |
| 1. Treatment #c. Wealth | 0.78* | (0.60 - 1.02) |
| 1.Time | 2.15 | (0.71 - 6.53) |
| 0b. Time #co. Wealth | 1.00 | (1.00 - 1.00) |
| 1.time1#c. Wealth | 0.84 | (0.62 - 1.13) |
| 0b. Treatment #0b. Time | 1.00 | (1.00 - 1.00) |
| 0b. Treatment #1o. Time | 1.00 | (1.00 - 1.00) |
| 1o. Treatment #0b. Time | 1.00 | (1.00 - 1.00) |
| 1. Treatment #1. Time | 1.00 | (0.24 - 4.12) |
| 0b. Treatment #0b. Time #co. Wealth | 1.00 | (1.00 - 1.00) |
| 0b. Treatment #1o. Time #co. Wealth | 1.00 | (1.00 - 1.00) |
| 1o. Treatment #0b. Time #co. Wealth | 1.00 | (1.00 - 1.00) |
| 1. Treatment #1. Time #c. Wealth | 1.23 | (0.84 - 1.78) |
| **Age group (compared with 15-19)** | | |
| 20-34 | 0.88 | (0.35 - 2.18) |
| 35-49 | 1.05 | (0.35 - 3.14) |
| **Marital Status (compared with Single)** | | |
| Married | 0.77 | (0.44 - 1.36) |
| **Education (compared with No formal education)** | | |
| Primary/JHS/Middle SCH | 0.97 | (0.66 - 1.43) |
| Secondary School+ | 0.82 | (0.39 - 1.71) |
| **Religion (compared with Christianity)** | | |
| Traditional African Religion | 1.18 | (0.85 - 1.62) |
| Islamic Religion | 0.96 | (0.65 - 1.40) |
| **Location of Residence (compared with Urban)** | | |
| Semi-urban | 1.78 | (0.76 - 4.21) |
| Rural | 1.61 | (0.76 - 3.42) |
| **Parity (compared with one birth)** | | |
| 2-4 births | 0.96 | (0.55 - 1.66) |
| 5 or more births | 1.17 | (0.60 - 2.27) |
|  |  |  |
| Constant | 4.25** | (1.35 - 13.43) |

**Table S2: Logistic Regression Results of the effect of Education on DTP3 Vaccination Coverage**

| **VARIABLES** | **OR** | **95% CI** |
| --- | --- | --- |
|  | | |
| Education | 0.91 | (0.58 - 1.42) |
| 1.Treatment | 0.73 | (0.26 - 2.06) |
| 0b. Treatment #co. Education | 1.00 | (1.00 - 1.00) |
| 1. Treatment #c. Education | 1.32 | (0.67 - 2.59) |
| 1.Time | 1.69 | (0.53 - 5.36) |
| 0b. Time #co. Education | 1.00 | (1.00 - 1.00) |
| 1. Time #c. Education | 0.81 | (0.45 - 1.46) |
| 0b. Treatment #0b. Time | 1.00 | (1.00 - 1.00) |
| 0b. Treatment #1o. Time | 1.00 | (1.00 - 1.00) |
| 1o. Treatment #0b. Time | 1.00 | (1.00 - 1.00) |
| 1. Treatment #1. Time | 1.38 | (0.28 - 6.90) |
| 0b. Treatment #0b. Time #co. Education | 1.00 | (1.00 - 1.00) |
| 0b. Treatment #1o. Time #co. Education | 1.00 | (1.00 - 1.00) |
| 1o. Treatment #0b. Time #co. Education | 1.00 | (1.00 - 1.00) |
| 1. Treatment #1. Time #c. Education | 1.20 | (0.47 - 3.07) |
| **Age group (compared with 15-19)** | | |
| 20-34 | 0.88 | (0.36 - 2.15) |
| 35-49 | 1.04 | (0.36 - 3.04) |
| **Marital status (compared with Single)** | | |
| Married | 0.77 | (0.43 - 1.37) |
| Wealth index (compared with Q1-poorest) | | |
| Poorer | 1.18 | (0.70 - 1.99) |
| Middle | 1.19 | (0.78 - 1.82) |
| Less poor | 0.96 | (0.58 - 1.60) |
| Least poor | 0.89 | (0.56 - 1.40) |
| **Religion (compared with Christianity)** | | |
| Traditional African Religion | 1.16 | (0.84 - 1.60) |
| Islamic Religion | 0.92 | (0.62 - 1.39) |
| **Location of Residence (compared with Urban)** | | |
| Semi-urban | 1.83 | (0.78 - 4.30) |
| Rural | 1.76 | (0.84 - 3.68) |
| **Parity (compared with one birth)** | | |
| 2-4 births | 0.95 | (0.55 - 1.66) |
| 5 or more births | 1.19 | (0.62 - 2.30) |
|  | | |
| Constant | 6.03*** | (1.73 - 21.07) |
